# Diploid males support a two-step mechanism of endosymbiont-induced thelytoky in a parasitoid wasp

**DOI:** 10.1186/s12862-015-0370-9

**Published:** 2015-05-12

**Authors:** Wen-Juan Ma, Bart A. Pannebakker, Louis van de Zande, Tanja Schwander, Bregje Wertheim, Leo W. Beukeboom

**Affiliations:** Evolutionary Genetics, Groningen Institute for Evolutionary Life Sciences, University of Groningen, Groningen, The Netherlands; Department of Ecology and Evolution, University of Lausanne, Lausanne, Switzerland; Laboratory of Genetics, Wageningen University, Wageningen, The Netherlands

**Keywords:** Diploid males, Diploidization, Endosymbiont, Feminization, Haplodiploidy, Sex determination, Thelytoky, *Wolbachia* titer

## Abstract

**Background:**

Haplodiploidy, where females develop from diploid, fertilized eggs and males from haploid, unfertilized eggs, is abundant in some insect lineages. Some species in these lineages reproduce by thelytoky that is caused by infection with endosymbionts: infected females lay haploid eggs that undergo diploidization and develop into females, while males are very rare or absent. It is generally assumed that in thelytokous wasps, endosymbionts merely diploidize the unfertilized eggs, which would then trigger female development.

**Results:**

We found that females in the parasitoid wasp *Asobara japonica* infected with thelytoky-inducing *Wolbachia* produce 0.7–1.2 % male offspring. Seven to 39 % of these males are diploid, indicating that diploidization and female development can be uncoupled in *A. japonica. Wolbachia* titer in adults was correlated with their ploidy and sex: diploids carried much higher *Wolbachia* titers than haploids, and diploid females carried more *Wolbachia* than diploid males. Data from introgression lines indicated that the development of diploid individuals into males instead of females is not caused by malfunction-mutations in the host genome but that diploid males are most likely produced when the endosymbiont fails to activate the female sex determination pathway. Our data therefore support a two-step mechanism by which endosymbionts induce thelytoky in *A. japonica*: diploidization of the unfertilized egg is followed by feminization, whereby each step correlates with a threshold of endosymbiont titer during wasp development*.*

**Conclusions:**

Our new model of endosymbiont-induced thelytoky overthrows the view that certain sex determination mechanisms constrain the evolution of endosymbiont-induced thelytoky in hymenopteran insects. Endosymbionts can cause parthenogenesis through feminization, even in groups in which endosymbiont-diploidized eggs would develop into males following the hosts’ sex determination mechanism. In addition, our model broadens our understanding of the mechanisms by which endosymbionts induce thelytoky to enhance their transmission to the next generation. Importantly, it also provides a novel window to study the yet-poorly known haplodiploid sex determination mechanisms in haplodiploid insects.

**Electronic supplementary material:**

The online version of this article (doi:10.1186/s12862-015-0370-9) contains supplementary material, which is available to authorized users.

## Background

Although sexual reproduction is ubiquitous in nature, approximately two percent of described insect species reproduce parthenogenetically [1, 2], including cyclical parthenogens (e.g., aphids and cynipids), facultative parthenogens, as well as obligate parthenogens. Thelytoky is a form of parthenogenesis in which unfertilized eggs develop into females. In insects, thelytokous reproduction can be determined by the host’s nuclear genome (e.g., [3–6]), but also by intracellular endosymbionts such as *Wolbachia*, *Cardinium* and *Rickettsia* (e.g., [7–9]). Endosymbionts are vertically transmitted via the egg cytoplasm and therefore not transmitted by males, thelytoky-induction is adaptive for the endosymbionts because it enhances their transmission rate. Endosymbiont-induced thelytoky has been mainly documented in haplodiploid species (reviewed in [10–12]). Under haplodiploidy, females are diploid and develop from fertilized eggs while haploid males develop from unfertilized eggs [2, 13]. Endosymbiont-infected females in these species lay unfertilized eggs that undergo diploidization and develop into females, but how these processes are induced and regulated remains enigmatic.

The simplest explanation of how endosymbionts could induce thelytoky in haplodiploids is that they cause haploid eggs to undergo diploidization. These diploidized eggs would then develop into females as a consequence of the host’s haplodiploid sex determination system [7, 8, 10, 14–18]. However, a recent study by Giorgini *et al.* [9] found that curing the parasitoid wasp *Encarsia formosa* of its thelytoky-inducing *Cardinium* with antibiotics resulted in progenies consisting entirely of diploid males, rather than haploid males as expected under haplodiploidy*.* A likely explanation is that *Cardinium* in this species induces the feminization of diploid embryos, whereas the diploidization of unfertilized eggs is controlled by the host’s genome [9]. Diploid males were also detected in the progenies of infected thelytokous *Trichogramma* wasps that had lowered *Wolbachia* titer following heat treatment [19]. These two studies suggest that female development under endosymbiont-induced thelytoky is not necessarily attained through diploidization alone, and that feminization may not be a direct consequence of diploidization.

Here, we investigate the causes of the frequent and spontaneous occurrence of males (haploid and diploid) in *Wolbachia*-infected thelytokous populations of the parasitoid wasp *Asobara japonica* [20, 21], and use this information to propose a model for *Wolbachia*-induced thelytokous reproduction. First, we quantified the frequency of haploid and diploid males among four thelytokous strains. Next, we examined the effect of the *Wolbachia* titer of parental females on the frequency and ploidy of males among their progenies, because *Wolbachia* dosage-dependent effects have been implicated in male production in other thelytokous species (e.g., [22–25]). We further assessed the association between *Wolbachia* titer and ploidy and sex of individual wasps. Finally, we introgressed alleles from a thelytokous into a sexual strain, to test whether the production of diploid males could be due to malfunction-mutations in the thelytokous genome for the female sex determination pathway. Such mutations could render individuals with a thelytokous genome unable to undergo feminization in the absence of the endosymbiont. Long-term infection with endosymbionts can result in a dependency of the host on its symbiont, for processes formerly accomplished by pathways encoded by the host genome (e.g., [26, 27]). Such a dependency may occur for female development in species with endosymbiont-induced thelytoky. Since it is the endosymbiont that triggers female development, females cured of their endosymbionts always produce sons, [14–16, 21]. Our previous study has ruled out complementary sex determination for *A. japonica* [28], but it is currently not known what its sex determination mechanism is (see Discussion for further details).

Our results suggest that diploidization and feminization are two separate processes required for *Wolbachia*-induced thelytoky in *Asobara japonica*, and that these two processes may depend on different *Wolbachia* titers. The combination of these findings with the studies of Giorgini *et al.* [9] and Tulgetske [19] led us to formulate a two-step model for the mechanism of endosymbiont-induced thelytoky. We discuss how this model challenges the view that endosymbiont-induced thelytoky can only occur in haplodiploid species with specific molecular mechanisms of sex determination.

## Methods

### Wasp cultures and male frequency

Four *Wolbachia*-infected thelytokous strains and one uninfected sexual strain of *Asobara japonica* were used for investigating the causes of male development under thelytoky*.* All strains were collected in Japan, described in Mitsui *et al.* [29] and Murata *et al.* [30], and kindly provided by M.T. Kimura. The sexual strain originated from the island of Amami-oshima (AO), and the four thelytokous strains were originally collected from Sapporo (SPP), Kagoshima (KG), Hirosaki (HR) and Tokyo (TK) on the mainland of Japan. Wasps were cultured on second-instar *Drosophila melanogaster* larvae as hosts at 25 °C, with a 16L: 8D light–dark cycle and 60 % relative humidity (for details see [28]).

To investigate the frequency of male production in different thelytokous strains, three replicates each consisting of five to eight females from all four thelytokous strains were offered approximately 200 second-instar *D. melanogaster* larvae for oviposition. The emerged offspring were counted and sexed. This procedure was repeated for five successive generations for the thelytokous KG strain to obtain sufficient sample sizes for the introgression experiment (see below).

### Determination of ploidy level

Flow cytometry was used to determine the ploidy level of males (n = 298) among progenies of females from the four thelytokous strains, following methods described by Ma *et al.* [28]. In short, the head of each individual (freshly killed by freezing at -20 °C) was homogenized in 500 μl Galbraith buffer and the DNA was stained with 10 μl propidium iodide (2.5 mg/ml). The total DNA content of approximately 2500 nuclei was measured on a Coulter Epics MXL flow cytometer (Beckman Coulter, Miami, FL, USA), or 10,000 nuclei on BD FACSAria™ II Cell Sorting System (Becton Dickinson B.V., Breda, The Netherlands). Flow cytometric DNA histograms of sampled wasp individuals were generated with WinMDI software package (version 2.9, The Scripps Research Institute, La Jolla, CA, USA) or BD FACSDiva™ (version 6.1.2; see Additional file 1: Figure S1). Two females per strain (collected from mass cultures) were used as diploid controls. Males were classified as haploid or diploid by comparing the mean log-transformed propidium iodide fluorescence (representing DNA amount per nucleus) to the diploid control.

### Antibiotic treatment

To test whether the *Wolbachia* titer of a parental thelytokous female affects male offspring production, different concentrations of the antibiotic rifampicin (Sigma-Adlrich, St. Louis, USA) were fed to thelytokous HR females. Rifampicin has been shown to have little impact on the development of *Asobara* wasps [26]. No effect on life-history traits, such as brood size and pupal mortality, was found in our experiments (data not shown). HR thelytokous females were offered host larvae that had fed on a yeast suspension containing different concentrations of rifampicin ranging from 0 (control), 0.00001, 0.00005, 0.0001, 0.001, 0.005, 0.01, 0.05, 0.1 and 1 mg/g (RIF0-RIF9). Wasp larvae ingest the antibiotics by feeding on the treated hosts and differentiate into females with a reduced endosymbiont titer [26]. Ten females were randomly selected from each antibiotic concentration. Each female was allowed to lay eggs in approximately 50 untreated host larvae, and offspring emerging from these hosts were counted and sexed (data are available at Dryad repository [31]). *Wolbachia* titers were determined via quantitative PCR (see below) both in the thelytokous mothers and in a randomly collected set of males and females from the mass culture.

### Quantitative PCR (qPCR)

The effect of antibiotic treatment on *Wolbachia* titer of females (emerged from the treated host larvae) was quantified by qPCR in three females per applied antibiotic concentration [31]. Similar qPCRs were performed to establish the correlation between *Wolbachia* titer and ploidy and sex of wasps from the mass culture [31] (females: n = 18, haploid males: n = 12, diploid males: n = 12). Genomic DNA was extracted from the whole body of each adult wasp individual, using a high-salt protocol (adjusted from [32]). The *Wolbachia* titer was determined from the amount of the *Wolbachia*-specific *gatB* gene, and two *A. japonica* reference genes, the nuclear *ITS2* (internal transcribed spacer) and mitochondrial *COI* (cytochrome oxidase I subunit, see Additional file 2: Figure S2). The following primers were used (from [20, 33]): *gatB*-F: 5′-GAA GCA AAG AGG ATG CAA GC-3′ and *gatB*-R: 5′-TCC TGG CTT ACC TCA ACA GG-3′, producing a 73-bp amplicon; *ITS2*-F: 5′-GGC AAG CAC AAT CAA GGT CT-3′ and *ITS2*-R: 5′-ACA AAA ACA AAT TTT GCG GC-3′, producing a 93-bp amplicon; *COI*-F: 5′-ACC TGT AAT ATT AGG TGG ATT TGG-3′ and *COI*-R: 5′-CCA ACA CCT ACA TTT AAT ATT CCT CT-3′, producing a 139 bp amplicon. Each qPCR was performed in a total volume of 25 μl, containing 1× SybrGreen Mastermix (Warrington, Cheshire, UK), 400nM forward primer, 400nM reverse primer and 2 μl DNA template. The Applied Biosystems 7300 Real-Time PCR System (Applied Biosystems Inc., Singapore) was used for all qPCR reactions. qPCR conditions for all genes were as follows: 15 min at 95 °C, then 40 cycles of 25 s at 95 °C, 45 s at 52 °C and 1 min at 72 °C, and finally 7 min at 72 °C.

The qPCR data were analysed with the LinRegPCR software (version 2012.3.2.0, [34, 35]) to calculate the starting concentrations (N_0_) of the target and the reference genes [36]. The efficiency of primers and PCR was optimized prior to the qPCR. The relative quantity of *gatB* was established as the ratio (R) of the starting concentrations of the focal gene (N_0__*gatB*) to the geometric mean of the reference genes (N_0__*COI*&*ITS2*): R = N_0__*gatB/* N_0__*COI*&*ITS2*.

### Introgression experiment

**Fig. 1:**
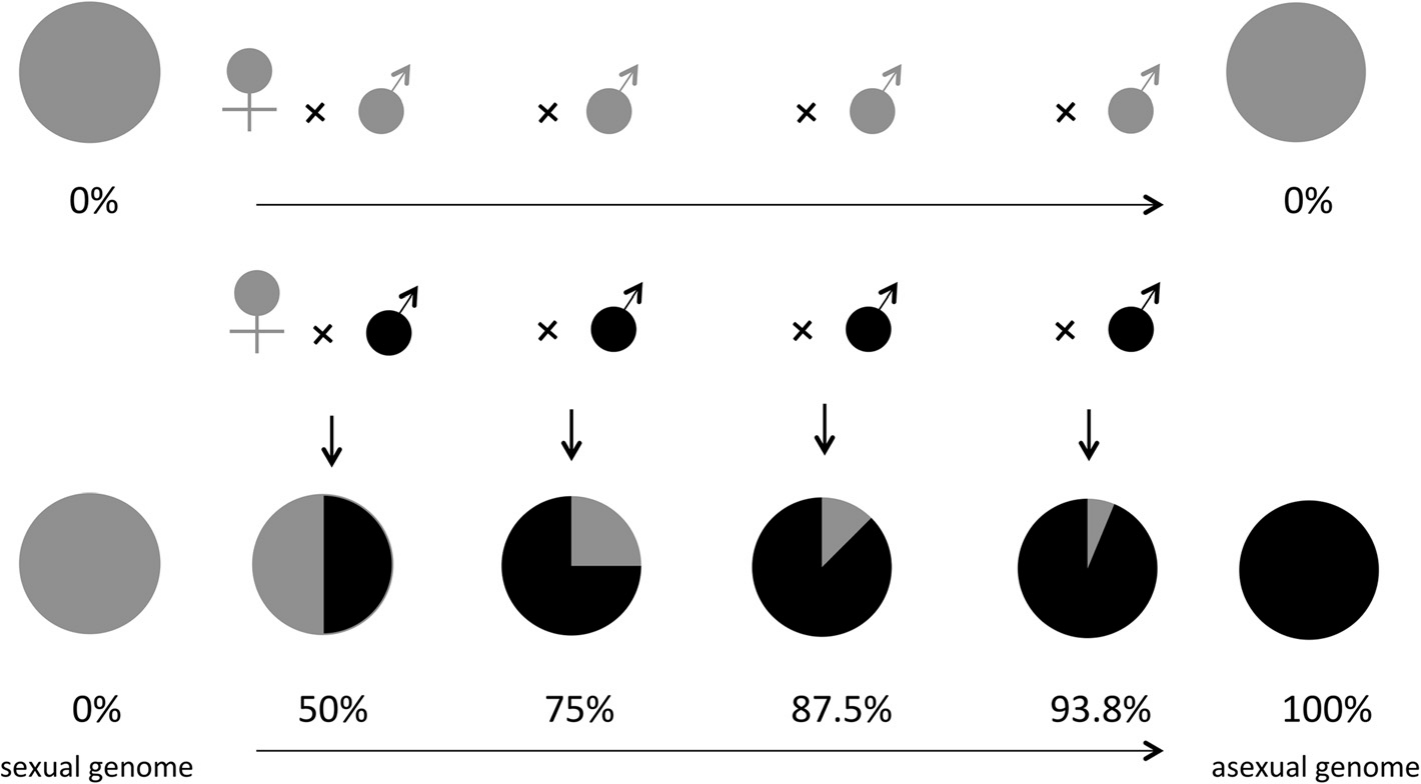
Introgression experiment. Sexual females were backcrossed to asexual males for four generations. Grey & black symbols depict sexual and asexual genotypes respectively. Pie charts show the relative asexual composition of the female genome for progressive generations of introgression

To test whether individuals with a thelytokous genome were unable to undergo feminization in the absence of the endosymbiont, alleles of the thelytokous KG strain were introgressed into the sexual AO genome (Fig. 1). AO females that were mated with AO males served as a sexual control (data from [21]). The introgression started by setting up crosses between males from the KG strain with females from the sexual AO strain for 24 h. Spontaneously occurring asexual KG males may be haploid or diploid. Diploid males were shown to be fertile, although they produce a lower number of female offspring (triploid) than haploid males [37]. As triploid females were found to be sterile, we only selected haploid KG males as fathers in the introgression experiment (the ploidy of males was determined after the matings and matings involving diploid males were discarded). Sixty sexual females mated to haploid asexual KG males were subsequently placed in a glass bottle with agar medium and a layer of 1.5 ml yeast solution (0.4 g/ml) containing approximately 100 second-instar host larvae for oviposition during 36 h (for details see [28]). After approximately 13 days, male offspring emerged and were collected (males emerge earlier than females). The remaining wasp pupae were individually isolated in vials to obtain virgin hybrid females. These F1 hybrid females were backcrossed to a thelytokous KG male (with haploidy verified as described above). This experimental procedure was repeated for four generations, yielding females with 50 % (hybrid introgression generation 1, F1), 75 % (backcross introgression generation 2, G2), 87.5 % (G3), and 93.8 % (G4) of the thelytokous genome (Fig. 1). Brood sizes and the proportions of female offspring were scored per generation (due to logistic reasons, phenotyping of the G3 generation was not possible and therefore no data are provided), with sexual AO females mated with AO males serving as a control [31] (data from [21]). As the proportion of hybrid females that produced daughters (i.e., were still able to mate and fertilize their eggs) decreased during successive introgression generations [21], the number of broods with at least one daughter that could be used for offspring sex ratio analysis decreased gradually. Thus, the number of broods used for sex ratio analysis decreased from 60 in the cross between sexual females to KG males, to 8 in the F1 generation, to 8 in G2 and 4 in G4. The ploidy of 51 randomly selected male offspring produced during F1 generation was analysed by flow cytometry (as described above). The ploidy of male offspring from subsequent generations was not analysed, because offspring sex ratios in these generations indicated that the ratio of diploid to haploid males did not vary (see Results for details).

### Statistical analyses

A generalized linear model (glm) was used to test possible effects of antibiotic treatment of parental females on male offspring production. Antibiotic concentration was used as a quantitative explanatory variable and the proportion of male offspring as the response variable (weighted by the brood size via the logit function), with a quasi-binomial error structure to correct for over-dispersion [38]. To compare diploid male numbers between antibiotic treatments, a glm was used in which the number of diploid males was the response variable and antibiotic concentration a qualitative explanatory variable with a log link function and a quasi-poisson error structure to correct for over-dispersion [38]. Multiple comparisons of diploid male numbers among different antibiotic treatments were performed with the Tukey test, as implemented in the R package multcomp for general linear hypotheses [39].

A glm was used to compare the *Wolbachia* titer among females of the various antibiotic treatments, measured as the gene quantity of *gatB* relative to the endogenous controls. As *Wolbachia* titers were normally distributed, this glm used an identity link function and a gaussian error structure. *Wolbachia* titer was used as the response variable and the antibiotic concentration as a quantitative explanatory variable. To test whether *Wolbachia* titer differed between individuals with different ploidy levels, diploids (females and males) were compared with haploid males using a Wilcoxon rank-sum test. The *Wolbachia* titer between diploid females and diploid males was analysed in a similar way.

Glms were also used to compare brood sizes and daughter proportions among introgression generations, with the percentage of the thelytokous genome as a quantitative explanatory variable. Brood size was modeled as the response variable with a quasi-poisson error structure to correct for over-dispersion, and proportion of female offspring as the response variable with a quasi-binomial error structure to correct for over-dispersion [38]. To check for the possibility of hybrid breakdown causing male mortality during the introgression experiment, male offspring numbers from virgin sexual and virgin introgressed F1 (50 % sexual–50 % thelytokous) females were compared with a glm model. In this glm, female genotype is the explanatory variable and the male offspring number the response variable with a quasi-poisson error structure to correct for over-dispersion [38].

## Results

### Frequency and ploidy of males

Males were found at frequencies of 0.7 %, 1.2 % and 1.2 % (n = approximately 2,500 offspring per strain) among the progenies of females from the thelytokous strains SPP, HR and TK, respectively. Similarly, a total of 220 males (1.2 %) were found in the KG strain using a much larger sample size (18,000 offspring; Table 1). These male frequencies in thelytokous *A. japonica* are similar to the ones reported by Reumer *et al*. [20]. Flow cytometry revealed that 73 % of these males (218 out of 298) were haploid and the remaining 27 % (80 out of 298) were diploid, with proportions of diploid males ranging from 7-39 % among different thelytokous strains (Table 1).

**Table 1:** Number and ploidy level of males in progenies of thelytokous females of four different A. japonica strains

Line	No. haploid males	No. diploid males	No. total males	Prop. diploid males
SPP	11	7	18	39 %
KG	153	67	220	30 %
HR	26	4	30	13 %
TK	28	2	30	7 %
Total	218	80	298	27 %

### Effect of *Wolbachia* titer

**Fig. 2:**
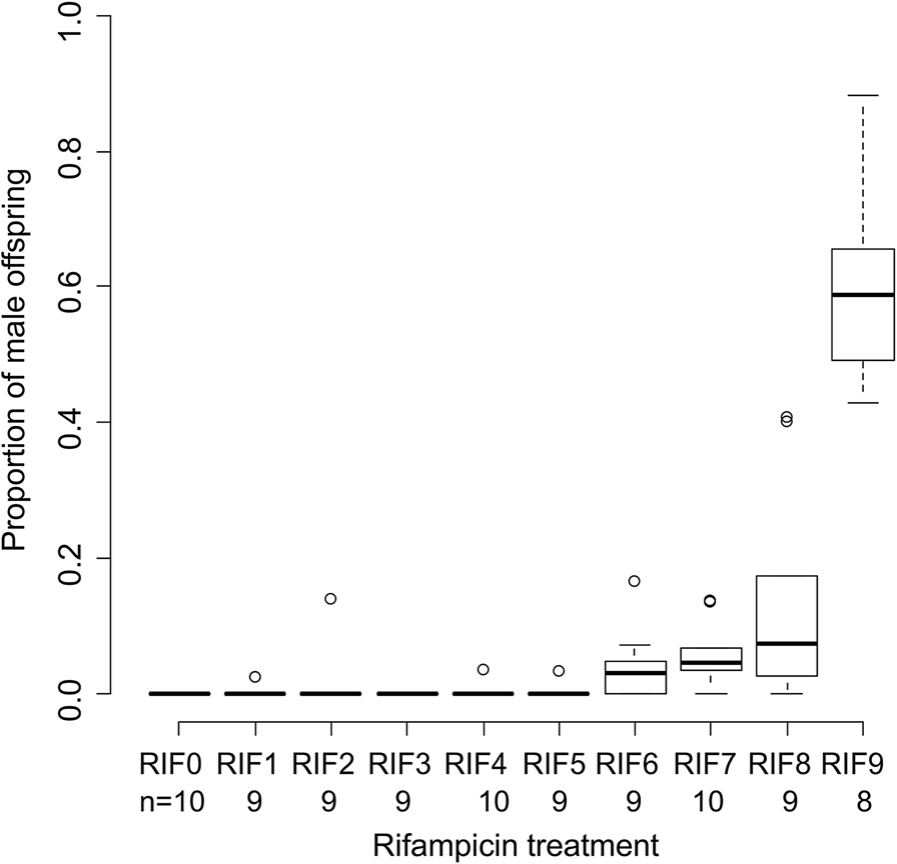
Proportion of males among progenies of thelytokous HR strain females treated with different concentrations of rifampicin (RIF). RIF0 is the no antibiotics treatment control, RIF1 to RIF9 depict the series of different concentrations of rifampicin (0.00001, 0.00005, 0.0001, 0.001, 0.005, 0.01, 0.05, 0.1 and 1 mg/g respectively). Sample sizes (n) are given for each treatment

**Fig. 3:**
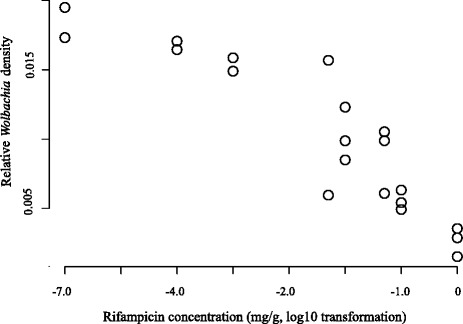
*Wolbachia* titer in parental females as a function of rifampicin (RIF) treatment. *Wolbachia* titer is determined by qPCR of the *gatB* gene relative to the reference genes *COI* and *ITS2*, and depicted along the x-axis as log transformed values

To investigate whether male production depended on *Wolbachia* titer of parental females, thelytokous females were offered host larvae that had been fed different antibiotic concentrations. The vast majority of emerged wasps from these antibiotics-treated hosts were females (approximately 98.7 %). For each antibiotic treatment, ten females were randomly selected and were then offered untreated host larvae for oviposition and their offspring were counted and sexed. The proportion of male offspring (haploid and diploid combined) gradually increased with higher concentrations of antibiotics administered to the hosts, ranging from 0 % to approximately 60 % (glm, F_1,91_ = 158.88, *P* < 0.0001, Fig. 2). *Wolbachia* titer in mothers was verified by qPCR and revealed that higher antibiotic concentrations indeed resulted in lower *Wolbachia* titers (glm, F_1,19_ = 63.70, *P* < 0.0001; Fig. 3). Diploid males occurred at very low frequencies among the progeny of all antibiotic-treated thelytokous females, and there was no significant difference among antibiotic treatments in the number of diploid males (glm, F_9,91_ = 0.916, *P* = 0.341; Table 2).

**Table 2:** Number of haploid and diploid males among progenies of thelytokous HR strain females treated with different antibiotic concentrations

Treatment	RIF0	RIF1	RIF2	RIF3	RIF4	RIF5	RIF6	RIF7	RIF8	RIF9
Haploid males	0	2	4	0	0	1	6	13	13	21
Diploid males	0	0	0	0	1	0	0	2	1	1
Total tested	0	2	4	0	1	1	6	14	14	22

RIF = rifampicin, see text for concentrations

**Fig. 4:**
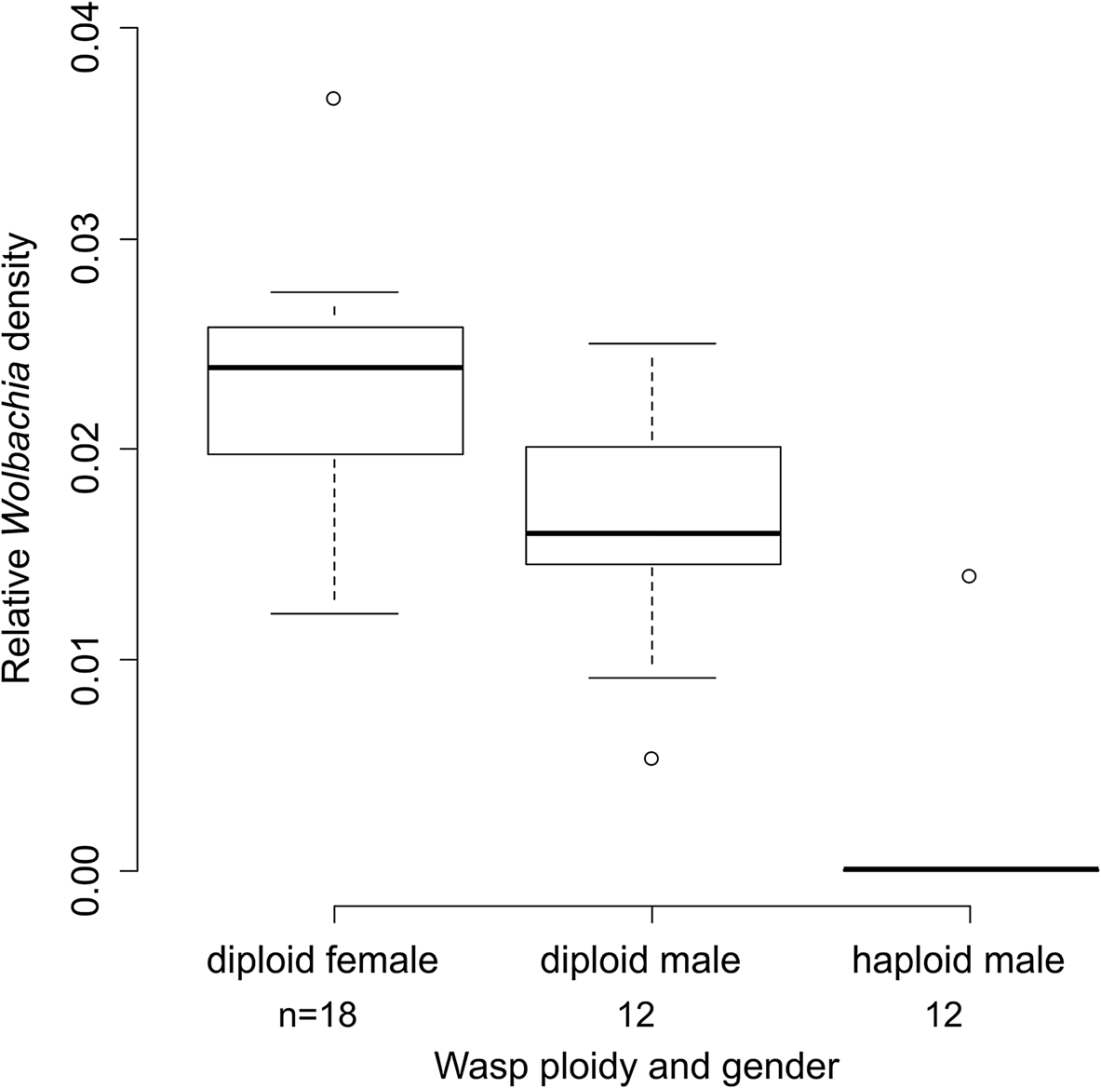
*Wolbachia* titer in diploid females, diploid males and haploid males in the untreated thelytokous KG strain. *Wolbachia* titer is determined by qPCR of the *gatB* gene relative to the reference genes *COI* and *ITS2.* Sample sizes (n) are given for each treatment

We then assessed the association between *Wolbachia* titer and ploidy, and between *Wolbachia* titer and the sex of thelytokous offspring: haploid males, diploid males and diploid females. All tested diploid females and diploid males were infected by *Wolbachia*. By contrast, only 7 out of 12 of haploid males were infected. The *Wolbachia* titer of diploid individuals (females and males pooled) was significantly higher than that of haploid males (Wilcoxon test, *W* = 5, *P* < 0.0001; Fig. 4), suggesting that a minimum *Wolbachia* titer is required to cause diploidization of unfertilized haploid eggs. Moreover, among the diploid individuals, the *Wolbachia* titer of females was significantly higher than that of males (Wilcoxon test, *W* = 59, *P* = 0.0088; Fig. 4), further suggesting an additional role for *Wolbachia* titer in the feminization of diploid embryos.

### *Wolbachia* effect on host sex determination

**Fig. 5:**
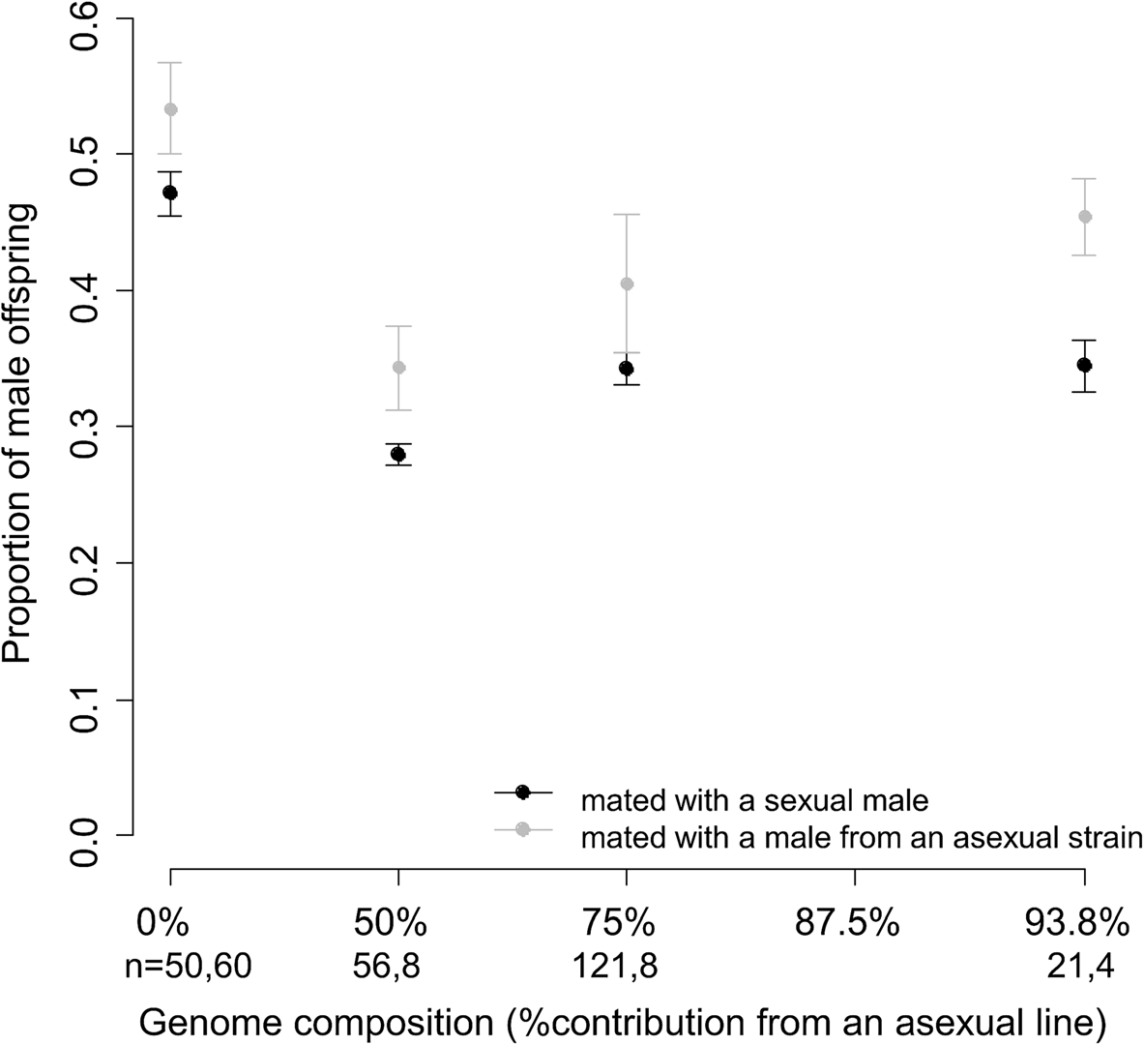
The proportion of male offspring in progenies of females with at least one daughter (i.e., females producing only sons are excluded) for different categories of sexual-asexual hybrid females and pure sexual females (100 % sexual genome). Females are mated with males from the thelytokous KG (in grey) or the sexual AO strain (in black, as a control, data from Ma *et al*. [14]). Bars indicate standard errors, *n* indicates the sample size for each generation and F1, G2, and G4 refer to the progressive introgression generations

We hypothesized that if the thelytokous host’s female sex determination pathway became dependent on *Wolbachia*, replacing the sexual genome by the thelytokous genome in the absence of *Wolbachia* may cause diploid fertilized eggs to develop into males rather than females. Upon introgression, diploid males are expected to develop from fertilized eggs of females from the first introgression generation onward if putative malfunction-mutations in the female sex determination pathway are dominant, and if such mutations are recessive, an increasing proportion of diploid males is expected when more thelytokous alleles are introgressed. The production of diploid sons would then generate a corresponding decrease of the proportion of daughters, since some fraction of fertilized eggs would develop into diploid males instead of females. However, we did not find an increase in diploid males, or a decrease in the production of daughters, across introgression generations. Not a single diploid individual was found in a random sample of 51 males from the F1 introgression generation and daughter production increased across introgression generations. Females of generations F1 and G2 produced a higher proportion of daughters than sexual controls when mated with a male from the thelytokous KG strain (glm, F1 and G2: *P* < 0.009; Fig. 5), and a higher proportion of female offspring was also found when these females had mated with sexual AO males (glm, Turkey contrast, all *P* < 0.001). These high proportions of female offspring are unlikely to stem from mortality of males as a consequence of hybrid breakdown because all-male broods of virgin sexual females and F1 introgressed females were equal in size (82.3 ± 2.2 versus 82.0 ± 3.0 males; glm, F_1,139_ = 0.006, *P* = 0.94). In addition, females of generation G4 with the highest representation of the thelytokous genome (estimated 93.8 %) produced a similar proportion of daughters as the control sexual cross (glm, t = −0.172, *P* = 0.863). These combined data provide evidence that the host sex-determination pathway did not become dependent on *Wolbachia*, and is not responsible for diploid male occurrence in the asexual strain.

## Discussion

Males are rare or absent in the majority of thelytokous species (reviewed in [40]). However, in agreement with previous reports [20, 33], we observed the frequent occurrence of males (0.7-1.2 %) in four thelytokous strains of the parasitoid wasp *A. japonica*. We found that a significant proportion (7-39 %) of these males are diploid rather than haploid as would be expected given their sex determination mechanism. We investigated the possible causes for this high occurrence of males (both haploid and diploid) to develop insights into how *Wolbachia* induces thelytoky in its host.

The simplest mechanism for endosymbiont-induced thelytoky in species with haplodiploid sex determination is that all-female progenies are the result of diploidization of unfertilized haploid eggs through the action of the endosymbiont (e.g., [14, 15, 41]). Diploid eggs would then develop into females following the host’s sex determination system. However, the spontaneous occurrence of diploid males in the progeny of thelytokous females (e.g., [19], this study), as well as upon removal of the endosymbiont [9] suggests that the mechanisms of thelytoky induction by endosymbionts may be more complex (i.e., involve feminization in addition to diploidization). Occasional development of haploid males in thelytokous strains has been considered to be the result of stochastic losses of the bacteria, (partial) failure of endosymbiont reproductive manipulation, or maladaptation between two co-evolved parties [20, 42]. However, how diploid males can arise in the broods of thelytokous females has thus far remained unexplained.

One possible explanation for the diploid male occurrence is that female sex determination has become dependent on the endosymbiont in thelytokous *A. japonica*. This has been observed in the lepidopterans *Ostrinia scapulalis* [43] and *Eurema mandarina* [44]. However, our introgression of alleles from a thelytokous into a sexual strain did not result in any diploid males, but led to higher proportions of females among the progenies in multiple generations of introgression. These results indicate that diploid males are not produced because of a defective female sex determination pathway in the thelytokous genomes. This implies that diploid males are most likely produced when *Wolbachia* fails to properly manipulate host reproduction.

We found an effect of *Wolbachia* titer in parental females on male production (haploids and diploids pooled), as exposing females to increasing antibiotic concentrations resulted in an increased production of males. Similar dosage-dependent effects of *Wolbachia* were found on male production in the thelytokous parasitoid *Muscidifurax uniraptor* [23], on cytoplasmic incompatibility in *Drosophila* [22], and on feminization in the leafhopper *Zyginidia pullula* [24] and the mosquito *Aedes aegypti* [25]. However, we did not find a similar *Wolbachia* dosage effect in parental females on the production of diploid males, although the probability of detecting such an effect was low given that we found few diploid males overall. A putative correlation between a mother’s *Wolbachia* titer and her propensity to produce diploid sons may also be weak because of the stochasticity in *Wolbachia* titers in individual eggs. Consistent with this view, we did find a correlation at the individual level between *Wolbachia* titer and wasp ploidy. Haploid males were often not infected at all, or, when infected, they carried a much lower *Wolbachia* titer than diploid individuals. This suggests that a minimal *Wolbachia* titer is required for the diploidization of unfertilized haploid eggs. Furthermore, diploid females carried significantly more *Wolbachia* than diploid males, suggesting an additional effect of *Wolbachia* density on the sex of diploid wasps. However, since the association of *Wolbachia* titers with ploidy and sex is correlational, other explanations, including differential *Wolbachia* proliferation in female ovaries vs male testes [45], cannot be excluded. An explicit test would require manipulation of *Wolbachia* titers in eggs or early embryos (after sex is determined), which is difficult to perform in practice. Note also that we implicitly assume that the measured *Wolbachia* titer in adults is proportional to the titer in their embryonic stage, when the endosymbiont acted on host sex determination. This may be a valid assumption given that Landmann *et al.* [45] found in nematodes that *Wolbachia* density does not change much during embryonic and early larval developmental stages but proportionally increased with age towards the adult stage.

**Fig. 6:**
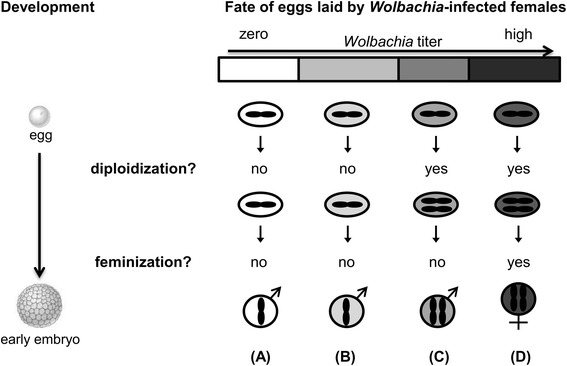
A two-step model for how endosymbionts induce thelytoky in haplodiploid species. Endosymbionts are considered to induce female development via two distinct steps: diploidization of the egg followed by feminization of the embryo. Each step relies on a certain threshold of endosymbiont titer during early embryonic development when sex is determined. There are four scenarios: **(A)** Absence of endosymbionts leads to haploid male development; **(B)** Low endosymbiont titer fails to initiate both diploidization and feminization, leading to development of haploid males as well; **(C)** Intermediate numbers of endosymbionts trigger diploidization, but are insufficient to induce feminization and result in diploid males; **(D)** High numbers of endosymbionts cause both diploidization and feminization, leading to diploid female development. Grey shading indicates endosymbiont titer, ranging from zero (white) to high (black)

Studies of Giorgini *et al.* [9] and Tulgetske [19] have already implied that diploidization and feminization are separate, uncoupled mechanisms in endosymbiont-induced thelytoky. Our results on thelytokous *A. japonica* corroborate this notion and take it a step further to suggest a two-step mechanism for the induction of thelytoky by *Wolbachia* based on endosymbiont density (Fig. 6): diploidization of the unfertilized egg is followed by feminization, and each step critically relies on a threshold of endosymbiont titer*.* In this two-step model, the first step involves diploidization of the unfertilized haploid egg. Absence or a very low density of endosymbionts in eggs would result in haploid male development (Fig. 6A and B). A higher density would result in diploid zygotes that can either develop into males or females (Fig. 6C and D). The second step involves feminization of diploidized embryos, which requires a high *Wolbachia* titer. If this titer is too low, failure of feminization occurs and embryos develop into diploid males (Fig. 6C). Only a sufficiently high bacterial titer leads to successful diploidization of unfertilized haploid eggs and subsequent feminization of diploid embryos (Fig. 6D). Note that the window of bacterial titer inducing diploidization but not feminization is assumed to be narrow, given that diploid males are rare overall.

Given the evidence of diploidization and feminization being separate processes in *A. japonica* and two other thelytokous species, there are at least three ways in which endosymbionts could induce thelytoky in haplodiploids. First, the endosymbiont would cause diploidization of haploid eggs, leading to female development following the host’s haplodiploid sex determination. Absence or failure of the endosymbiont leads to haploid male development and diploid males cannot occur. This has been suggested to be the case for *Wolbachia*-induced thelytoky in *Leptopilina clavipes* [16, 41] and *Muscidifurax uniraptor* [15]. Second, the endosymbiont feminizes eggs, while egg diploidization is under host control. This appears to apply to *Cardinium*-induced thelytoky in *Encarsia hispida,* in which curing of the bacteria leads to diploid rather than haploid male progenies [9]. Finally our results show that *Wolbachia* causes both diploidization of unfertilized haploid eggs and subsequent feminization of diploid embryos in *A. japonica*. (Partial) curing of thelytoky-inducing endosymbionts can therefore lead to haploid males or diploid males, most likely depending on the individual bacterial load. In addition to *A. japonica*, this appears to be the case in thelytokous *Trichogramma kaykai* infected with *Wolbachia* [19]. Future work needs to clarify whether diploidization and feminization are typically separate processes controlled by the endosymbionts or whether diploidization alone is sufficient to induce thelytoky in some host species. The latter may be difficult to demonstrate experimentally. Indeed, the mechanisms used by the endosymbionts to regulate diploidization and feminization may be highly canalized in most hosts, which would prevent the production of diploid males and generate the false impression of thelytoky induced via diploidization alone when in fact endosymbionts are also regulating feminization. Finally, it is also possible that different types of thelytoky induction are associated with specific endosymbiont species, such as *Wolbachia*, *Cardinium* and *Rickettsia* (e.g., [46]).

The realization that endosymbionts can induce thelytoky via diploidization and feminization refines our perspective on the possible constraints imposed by the molecular mechanisms of host sex determination on endosymbiont-induced thelytoky in haplodiploids [21, 47–49]. Two empirically confirmed sex-determination mechanisms have been documented in the Hymenoptera, Complementary Sex Determination (CSD), reported from over 60 hymenopteran species across most major superfamilies [47, 49, 50], and maternal effect genomic imprinting sex determination [51], thus far only described for the parasitoid wasp *Nasonia* [52]. Under CSD, individuals hemi- or homozygous at one or several loci develop into males, whereas heterozygous individuals develop into females. Hence, CSD has been considered to be incompatible with endosymbiont-induced thelytoky in which egg diploidization occurs via gamete duplication, because homozygous diploid eggs would develop into males rather than females [50, 53]. However, this view has to be reconsidered given our findings that both diploidization and feminization are regulated by *Wolbachia* in *A. japonica*. Indeed even under CSD, thelytoky via gamete duplication can occur, given the active feminization of diploid eggs by the endosymbiont regardless of the allelic state at the CSD locus. The same holds for the second sex determination mechanism known in hymenopterans, maternal effect genomic imprinting. Under genomic imprinting sex determination, female development depends on activation of the *transformer* gene in the zygote by a *trans* factor that is likely transcribed from the parental genomes [52, 54]. This sex determination mechanism could constrain the evolution of thelytoky, as femaleness depends on a parentally contributed chromosome complement (for a detailed discussion see [11]). However, as for CSD, such constraints are released if endosymbionts regulate feminization in addition to diploidization.

Further studies are needed on the interaction between sex determination and endosymbiont-induced thelytoky in *A. japonica* and other species. This will help to clarify whether or not molecular sex determination mechanisms constrain endosymbiont-induced thelytoky in haplodiploids. They also provide new opportunities for improving our knowledge of the yet poorly-understood sex determination mechanisms in haplodiploid insects. Endosymbiont-induced thelytoky and host sex determination may be subject to tight co-evolution, where one party might have to overcome the constraints set by the other party. It is therefore not unlikely that a completely novel sex determination mechanism, other than CSD or maternal effect genomic imprinting has evolved in *A. japonica* and other species, as a result of *Wolbachia* infection pressure. Further studies that manipulate endosymbiont titer by antibiotic treatment in combination with measuring gene expression of known sex determination genes may be very informative on the mechanisms of sex manipulation by endosymbionts.

## Conclusions

In this study, we found regular occurrence of diploid males in a *Wolbachia*-induced thelytokous species. Different approaches were taken to understand the underlying causes of this interesting phenomenon. *Wolbachia* density in the adult stage was shown to be correlated with the sex and ploidy of individual wasps. An introgression experiment indicated that diploid males were caused by elements encoded by the thelytokous genome. These results led us to propose a two-step mechanism for explaining the occurrence of diploid males: during early embryo development the endosymbiont induces thelytoky via diploidization and feminization. Each step appears to rely on a specific *Wolbachia* density. This challenges the common view that molecular sex determination mechanisms constrain endosymbiont-induced thelytoky in haplodiploids.

## Availability of supporting data

Data for this study are available at Dryad repository doi: http://dx.doi.org/10.5061/dryad.sk805, and other datasets supporting the results of this article are provided with Additional files.

## Additional files

Additional file 1: Figure S1. Flow cytometric DNA-histograms of a representative (a) haploid male, (b) diploid male and (c) diploid female in the thelytokous KG strain of *A. japonica*. The y-axis depicts the number of nuclei, and the x-axis the fluorescence intensity on a log scale, which converts to ploidy as indicated with the n-value. An excitation wave length of 488 nm and a band pass filter of 585 nm were used to detect propidium iodide fluorescence. Additional file 2: Figure S2. PCR assay of *Wolbachia* infection status among haploid males, diploid males and diploid females of the untreated thelytokous KG strain of *Asobara japonica*, using *Wolbachia*-specific *wsp* gene primers.

## Abbreviation

CSD: Complementary sex determination
