# Supplementary figures and images for: Diploid males support a two-step mechanism of endosymbiont-induced thelytoky in a parasitoid wasp
Source: BMC Evol Biol. 2015 May 12;15:84. doi: 10.1186/s12862-015-0370-9 (PMC4456809; doi:10.1186/s12862-015-0370-9)

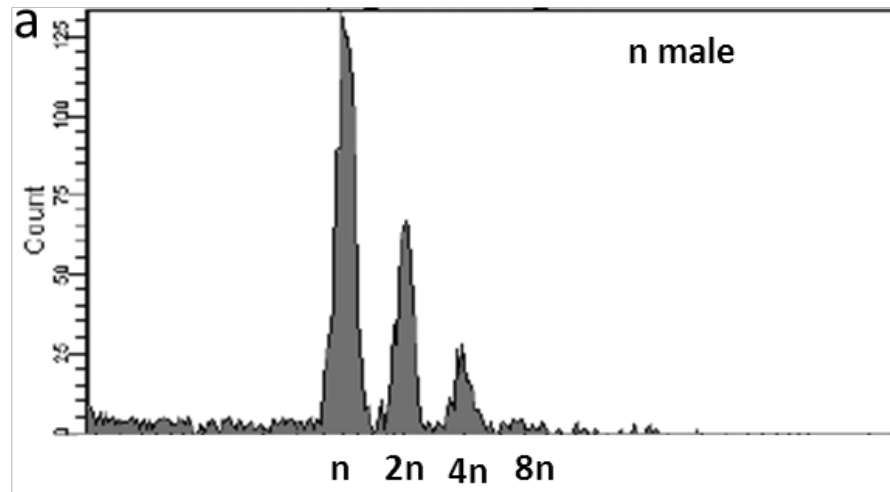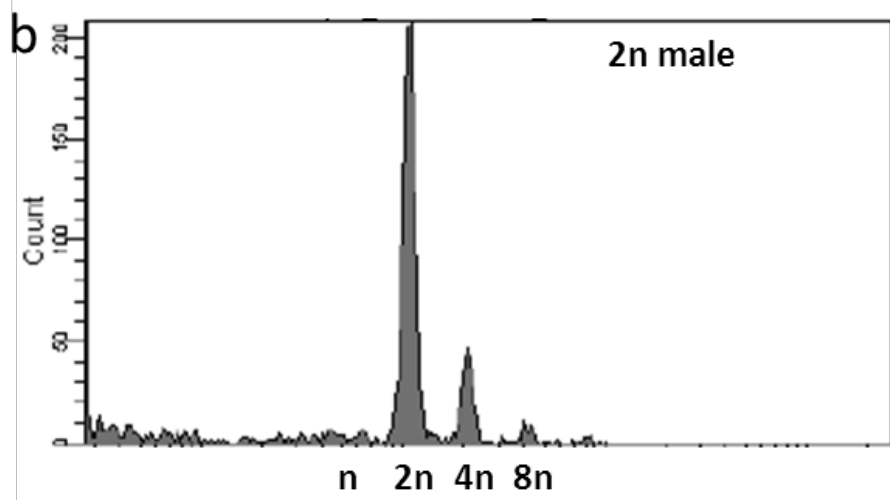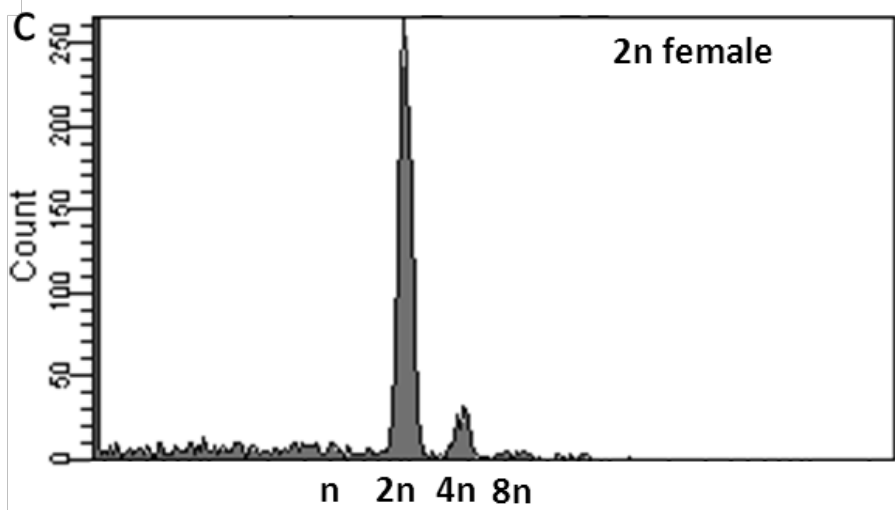

Supplement: Additional file 1: Figure S1. — Flow cytometric DNA-histograms of a representative (a) haploid male, (b) diploid male and (c) diploid female in the thelytokous KG strain of A. japonica. The y-axis depicts the number of nuclei, and the x-axis the fluorescence intensity on a log scale, which converts to ploidy as indicated with the n-value. An excitation wave length of 488 nm and a band pass filter of 585 nm were used to detect propidium iodide fluorescence. [file 12862_2015_370_MOESM1_ESM.pdf]

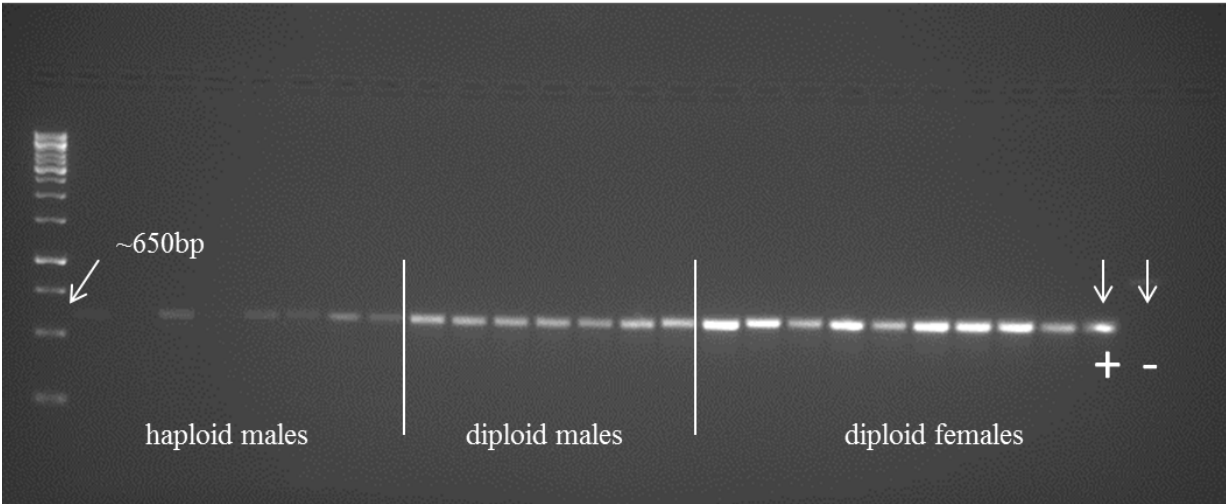

Supplement: Additional file 2: Figure S2. — PCR assay of Wolbachia infection status among haploid males, diploid males and diploid females of the untreated thelytokous KG strain of Asobara japonica, using Wolbachia-specific wsp gene primers. [file 12862_2015_370_MOESM2_ESM.pdf]
